# ADIPOQ rs2241766 SNP as protective marker against DIBC development in Mexican population

**DOI:** 10.1371/journal.pone.0214080

**Published:** 2019-03-18

**Authors:** Nelly Margarita Macías-Gómez, María Carmen Hernández-Terrones, Angélica Araceli Ramírez-Guerrero, Evelia Leal-Ugarte, Melva Gutiérrez-Angulo, Jorge Peregrina-Sandoval

**Affiliations:** 1 Departamento de Promoción, Preservación y Desarrollo de la Salud, Universidad de Guadalajara, Jalisco, México; 2 Facultad de Medicina e Ingeniería en Sistemas Computacionales de Matamoros, Universidad Autónoma de Tamaulipas, Tamaulipas, México; 3 Centro Universitario de Los Altos, Universidad de Guadalajara, Jalisco, México; 4 Centro Universitario de Ciencias Biológicas y Agropecuarias, Universidad de Guadalajara, Jalisco, México; Temple University School of Medicine, UNITED STATES

## Abstract

**Background:**

Adiponectin protein and some variations in its gene, *ADIPOQ* have recently been associated with cancer because they regulate glucose and lipid metabolism as well as anti-apoptotic and anti-inflammatory proteins.

**Aim:**

The aim of this study was to analyse the relationship between selected biochemical markers, anthropometric indices and *ADIPOQ* rs2241766 and rs1501299 SNPs in ductal infiltrating breast cancer (DIBC) in a Mexican population.

**Methods:**

This cross-sectional study included 64 DIBC patients and 167 healthy women. Polymerase chain reaction–restriction fragment length polymorphism (PCR-RFLP) analysis was performed to identify the genotypes of the rs2241766 (exon 2) and rs1501299 (intron 2) *ADIPOQ* polymorphisms. Corporal composition and biochemical markers included body mass index (BMI), waist circumference (WC), hip circumference (HC), waist-hip ratio (WHR), glucose, cholesterol, triglycerides and high- and low-density lipoprotein cholesterol.

**Results:**

Patients with DIBC had higher serum glucose, WC and WHR than controls. Intergroup differences in allele and genotype frequencies were found for both polymorphisms (*P* < 0.05). Patients carrying the rs2241766 TT and TG genotypes had higher values of WC, HC and WHR, but only TG carriers had higher levels of glucose. For the SNP rs1501299, carriers of the GG genotype in the DIBC group had higher values of glucose, WC, HC and WHR than the respective control group.

**Conclusions:**

These results suggest that WC, HC and WHR are better predictors of DIBC than BMI. The *ADIPOQ* SNP rs2241766 emerges as a protective factor, whereas rs1501299 is a risk factor for DIBC development in a Mexican population.

## Introduction

Breast cancer is the second most common malignancy in the world and the most common cancer in women. According to the International Agency for Research on Cancer (IARC) and the World Health Organization (WHO), there were 1.67 million new cases of breast cancer diagnosed around the world in 2012; a figure that represents 25% of all neoplasms [1]. Among distinct genetic and lifestyle factors, obesity seems to play a crucial role in breast cancer development. Adipose tissue secretes more than 50 bioactive factors, called “adipokines”, which exert endocrine and paracrine functions in diverse tissues. As one of the major adipocytokines, with plasma concentrations ranging from 3 to 30 ug/mL, adiponectin works to maintain the equilibrium between several physiological processes, such as serum glucose concentration, lipid metabolism and inflammation [2–8]. Recent studies have shown that variations in the adiponectin gene modulate serum concentrations of adiponectin, which are inversely associated with breast cancer and other neoplasms [9–14]. Approximately 2,511 variations have been identified in the human *ADIPOQ* gene, including the rs2241766 (exon 2) and rs1501299 (intron 2) single nucleotide polymorphisms (SNPs) highly associated with breast, colon, gastric, hepatocellular, prostate and endometrial cancers in different populations [9,13,15–21]. Among the diverse pathways regulated by adiponectin that are relevant to cancer, AMPK activation under glucose deprivation, hypoxia and oxidative stress emerges as the most beneficial, because it decreases cellular proliferation. Moreover, ADIPOQ is a potent inhibitor of the PI3K-mTOR pathway, either through phosphorylation of the protein raptor, a component of mTOR complex 1 (mTORC1), or by regulating the activity of tuberin (TSC2), which is a tumour suppressor. Other pathways influenced by ADIPOQ are those involving Wnt and leptin [22]. The aim of the current study was to analyse the association of the *ADIPOQ* SNPs rs2241766 and rs1501299 with the risk of ductal infiltrating breast cancer (DIBC) in a Mexican population and to assess the interaction between these alleles and corporal composition, lipid profile and plasma glucose concentration.

## Materials and methods

### Subjects

This cross-sectional study included 64 women with a histological diagnosis of DIBC and a control group of 167 healthy women with no familial history of breast cancer, who were randomly selected from the CUSUR university medical clinic in Ciudad Guzmán, Jalisco. Patients were enrolled from the RETO group in Ciudad Guzmán, Jalisco, Mexico, which is an association dedicated to the timely detection of breast cancer and to achieving the integral recovery of patients. All patients had undergone mastectomy 3 years (2007–2009) prior and were under chemotherapy or radiotherapy. Patients with metastasis, familiar history of breast cancer or any kind of cancer, diabetes mellitus type 2, arterial hypertension or any autoimmune disease where excluded of the study. The mean age of patients and controls was 46 ± 11 and 54 ± 8 years, respectively. The local ethics committee approved the protocol, with number 05-2010-1-853; in accordance with the Helsinki declaration and national guidelines, every woman enrolled in this study signed a consent form.

### Corporal composition determination

Body weight (kg) and height (cm) were measured with individuals wearing light clothing and no shoes, using a precision scale or a stadiometer. According to the body mass index (BMI), participants were classified as normal weight (<18.5–24.9 kg/m^2^), overweight (25.0–29.9 kg/m^2^) or obese (≥30.0 kg/m^2^) [23]. Waist circumference (WC) was measured at the umbilicus and was classified as no risk (<80 cm), low risk (80.0–87.9 cm) or high risk (>88.0 cm) [24]. Hip circumference (HC) was measured (cm) at the femoral greater trochanter. The subjects stood with arms at their sides and feet together for these two measures. As an additional measure of body fat distribution, we evaluated the waist-hip ratio (WHR), dividing the waist circumference by the hip circumference; subjects were classified as no risk (<0.85) or high risk (≥0.85) [25].

### Lipid and glucose profile determination

Glucose, cholesterol, triglycerides (TG), high-density lipoprotein cholesterol (HDLc), and low-density lipoprotein cholesterol (LDLc) concentrations were determined by colorimetric enzymatic procedures in serum from a 3 mL blood sample of each individual. The sample was taken after a minimum 8-h fast, immediately centrifuged at 1500 rpm at 4°C for 20 minutes and kept at −70°C until use. We used reagents supplied by SPINREACT laboratory and the *Microlab 200* microanalyzer spectrophotometer from MERK. Reference values were taken according the manufacturer as follows: for glucose, low (<60.0 mg/dl), normal (60.0–110.0 mg/dl) and high (>110.0 mg/dl); for cholesterol, normal (<200.0 mg/dl), borderline (200.0–239.0 mg/dl) and high (>240.0 mg/dl); for triglycerides, low (<35.0 mg/dl), normal (35.0–135.0 mg/dl) and high (>135.0 mg/dl); for HDLc, low risk (>65.0 mg/dl), normal risk (45.0–65.0 mg/dl) and high risk (< 45.0 mg/dl); and for LDLc, Optimal (<100 mg/dl), near optimal (100.0–129.0 mg/dl), Borderline high (130.0–160.0 mg/dl) and high (>160.0 mg/dl).

### Genotyping by PCR-RFLP

DNA was extracted from all individuals, using the CTAB-DTAB method, from the leukocytes of a 3 mL venous blood sample collected in tubes containing EDTA. In the case of women receiving chemotherapy, we obtained the sample 2 weeks after the most recent chemotherapy session. Genotypes were determined using the PCR-RFLP method previously reported [26]. A fragment of 305 bp of DNA containing the polymorphic site rs2241766 was amplified by polymerase chain reaction (PCR) using the forward primer 5´-TGT GTG TGT GGG GTC TGT CT-3´ and the reverse primer 5´-TGT GAT GAA AGA GGC CAG AA-3´. For the rs1501299 polymorphism, we amplified a 107-bp fragment by PCR using the forward primer 5´-CTA CAC TGA TAT AAA CTA TAT GGA G-3´ and the reverse primer 5´-CCC CAA ATC ACT TCA GGT TG-3´. After amplification, the fragments were digested with restriction enzymes *Ava*I (restriction site is created in the G allele) for rs2241766, and *Bsm*I (restriction site is lost in the T allele) for rs1501299 SNP. Fragments were separated by electrophoresis in a 15% polyacrylamide gel and visualized with AgNO_3_.

### Statistical analysis

Statistical analysis was performed with the SPSS package for Windows version 17.0. Descriptive statistics, namely means ± SD and the comparisons between the respective values in both groups, were evaluated using the Student’s *t-*test, and homogeneity of variance was assessed using Levene’s test. The distribution of allelic and genotype frequencies in both groups and the appropriate intergroup comparisons were assessed by chi-square analysis, whereas the association analysis was based on odds ratio (OR) and 95% confidence interval (CI) values. The criterion for statistical significance was *P* < 0.05.

## Results

Mean cholesterol concentrations were significantly higher in controls than in DIBC patients (*p* < 0.030); in contrast, glucose levels, WC, WHR and HC were higher in the latter (Table 1). The allele and genotype frequencies of both SNPs are shown in Table 2; notably, the respective allele frequencies in the control group were consistent with Hardy-Weinberg equilibrium. We observed significant differences between DIBC patients and control individuals for the TG (*P =* 0.02) and TG+GG (*P =* 0.01) genotypes in the rs2241766 SNP; similarly, we observed analogous differences for the TT (*P =* 0.003) and GT+TT (*P =* 0.027) genotypes of the SNP rs1501299 (Table 2).

**Table 1:** Anthropometric measures and lipid and glucose profile comparison between women in the control and DIBC groups.

Corporal composition measures	Total n = 231 (%)	DIBC n = 64 (%)	Control n = 167 (%)	P value
BMI (n = 231)	28.01±5.07	28.83±5.69	27.7±4.8	0.07
Normal	67 (29)	17 (26.6)	50 (29.9)	0.23
Overweight	93 (40.3)	22 (34.4)	71 (42.5)	
Obese	71 (30.7)	25 (39.1)	46 (27.5)	
WHR (n = 228)	0.85±0.627	0.88±0.06	0.84±0.061	0.00 a
No risk	103 (45.2)	19 (30.6)	84 (50.6)	0.007 a
High risk	125 (54.8)	43 (69.4)	82 (49.4)	
WC (n = 228)	88.51±12.04	94.74±11.86	86.2±11.3	0.00 a
No risk	55 (24.1)	6 (9.7)	49 (29.5)	0.002 a
Low risk	56 (24.6)	11 (17.7)	45 (27.1)	
High risk	117 (51.3)	45 (72.6)	72 (43.4)	
**Biochemical values**				
Glucose (mg/dL) (n = 195)	101.47±36.83	111.39±45.37	97.4±31.96	0.04 a
Low	5 (2.6)	2 (3.5)	3 (2.2)	0.120
Normal	146 (74.9)	37 (64.9)	109 (79)	
High	44 (22.6)	18 (31.6)	26 (18.8)	
Cholesterol, mg/dL (n = 228)	218.68 ± 57.17	205.15±61.43	223.62±184.25	0.027 a
Normal	82 (36)	26 (42.6)	56 (33.5)	0.03 a
Borderline	66 (28.9)	22 (36.1)	44 (26.3)	
High	80 (35.1)	13 (21.3)	67 (40.1)	
HDLc, mg/dL (n = 222)	50.46±22.03	51±27.55	50.26±19.7	0.82
Low risk	95 (42.8)	25 (41.7)	70 (43.2)	0.547
Normal risk	89 (40.1)	27 (45)	62 (38.3)	
High risk	38 (17.1)	8 (13.3)	30 (18.5)	
LDLc, mg/dL (n = 222)	135.18±55.13	128.1±43.28	137.8±58.82	0.18
Optimal	57 (25.7)	14 (21.9)	43 (26.5)	0.026 a
Near optimal	41 (18.5)	17 (26.6)	24 (14.8)	
Borderline high	48 (21.6)	16 (26.7)	32 (19.8)	
High	76 (34.2)	13 (21.7)	63 (38.9)	
TG, mg/dL (n = 228)	225.03±194.82	217.77±257.74	227.7±166.93	0.73
Low	1 (0.4)	0 (0)	1 (0.6)	0.165
Normal	76 (32.9)	27 (42.2)	49 (29.3)	
High	139 (66.7)	37 (57.8)	117 (70.1)	

Continuous variables are shown as mean ± SD; categorical variables are expressed as number of subjects and percentage. Independent variables were compared between control woman and those with DIBC by using the Student’s *t*-test and Chi-square test for continuous and categorical variables respectively.

^a^ Significance difference between groups considering P values (<0.05).

HDLc = High-density lipoprotein cholesterol, LDLc = Low-density lipoprotein cholesterol, TG = Triglycerides, WC = waist circumference, HC = Hip circumference, WHR = waist-hip ratio, BMI = Body mass index.

**Table 2:** Allele and genotype frequencies of *ADIPOQ* rs2241766 and rs1501299 polymorphisms in control women and those with DIBC.

**rs2241766**	**Control n = 167 (%)**	**DIBC n = 64 (%)**	**OR (95% CI)**	**P value**
T	276 (83)	119 (93)	1.0 (Reference)	
G	58 (17)	9 (7)	0.35 (0.17–0.75)	0.002^a^
TT x TT	115 (69)	55 (86)	1.0 (Reference)	
TG x TG	46 (27)	9 (14)	0.41 (0.18–0.89)	0.022^a^
GG x GG	6 (4)	0 (0)	0.16 (0.00–2.89)	0.23
TG +GG	52 (31)	9 (14)	0.36 (0.16–0.78)	0.01^a^
**rs1501299**	**Control n = 167 (%)**	**DIBC n = 64 (%)**	**OR (95% CI)**	**P value**
G	239 (71)	73 (57)	1.0 (Reference)	
T	95 (29)	55 (43)	1.84 (1.20–2.80)	0.002^a^
GG x GG	87 (52)	23 (36)	1.0 (Reference)	
GT x GT	65 (39)	27 (42)	1.5 (0.78–2.82)	0.16
TT x TT	15 (9)	14 (22)	3.4 (1.45–8.16)	0.003^a^
GT + TT	80 (48)	41 (64)	1.94 (1.07–3.5)	0.027^a^

Variables were compared with a chi-square test.

^a^ Represents a significant difference between the control and DIBC groups. *P* < 0.05.

We also analysed the possible association between rs2241766 and rs1501299 SNPs with some corporal and biochemical traits (Tables 3 and 4). In rs2241766 SNP, carriers of the TT or TG genotypes in the DIBC group had higher values of WC, HC and WHR, but only those with the TG genotype had higher levels of glucose (Table 3). Higher measures of WC, HC and WHR were observed for rs1501299 in DIBC patients. However, these differences were significant only for WC and HC in carriers of the GG or GT genotype and for WHR in carriers of both homozygous genotypes. Cholesterol and LDLc concentrations were higher in control subjects with the TT genotype, whereas increased glucose values were seen in DIBC patients with the GG genotype (Table 4).

**Table 3:** Comparison of corporal composition measures and biochemical determinations with rs2241766 genotypes.

rs2241766	TT			TG		
	Control n = 115	DIBC n = 55	P value	Control n = 46	DIBC n = 9	P value
Cholesterol (mg/dl)	225±56	208±61	0.070	218±55	286.25±62	0.145
HDLc (mg/dl)	51±21	48±3	0.401	48±18	48±17	0.372
LDLc (mg/dl)	142±58	130±41	0.123	124±62	130±41	0.725
TG (mg/dl)	229±166	229±273	0.985	213±169	229±273	0.248
Glucose (mg/dl)	96±29	107±43	0.087	102±40	106±43	0.029^a^
BMI (kg/m2)	28±5	29±6	0.363	27±4	30±5	0.089
WC (cm)	86±11	94±12	0.000^a^	86±12	94±12	0.005^a^
HC (cm)	102±9	107±11	0.004^a^	101±10	107±11	0.01^a^
WHR	0.84±0.06	0.88±0.06	0.001^a^	0.85±0.05	0.90±0.06	0.03^a^

Values are expressed in median ±SD. Values are expressed in mg/dl for TG = Triglycerides, HDLc = High-density lipoprotein cholesterol and LDLc = Low-density lipoprotein cholesterol. WC = Waist circumference, HC = Hip circumference, WHR = Waist-Hip ratio. Student’s *t*-test.

^a^ Represents significant difference between control and DIBC groups. *P* < 0.05.

**Table 4:** Comparison of corporal composition measures and biochemical determinations with rs1501299 SNP.

rs1501299	GG			GT			TT		
	Control n = 87	DIBC n = 23	P value	Control n = 65	DIBC n = 27	P value	Control n = 15	DIBC n = 14	P value
Cholesterol (mg/dl)	223±50	207±81	0.397	223±62	213±45	0.387	230±50	188±54	0.039^a^
HDLc (mg/dl)	49±20	57±41	0.433	50±19	48±16	0.576	56±19	48±20	0.309
LDLc (mg/dl)	129±57	117±52	0.370	144±61	139±37	0.651	162±49	124±39	0.035^a^
TG (mg/dl)	222±178	247±410	0.663	224±144	208±124	0.635	280±193	191±123	0.154
Glucose (mg/dl)	95±22	132±64	0.017^a^	84	84.8	1.0	95.1	100.9	0.921
BMI (kg/m2)	27±4	28±6	0.651	28±6	30±6	0.079	29±4	28±4	0.360
WC (cm)	86±11	95±13	0.001^a^	86±12	95±13	0.003^a^	89±10	94±9	0.198
HC (cm)	102±8	107±12	0.013^a^	102±11	109±13	0.006^a^	105±10	106±8	0.939
WHR (cm)	0.84±0.07	0.88±0.05	0.007^a^	0.84±0.06	0.87±0.06	0.075	0.85±0.03	0.89±0.06	0.031^a^

Mean values are expressed in mg/dl. TG = Triglycerides, HDLc = High-density lipoprotein cholesterol, LDLc = Low-density lipoprotein cholesterol. Student’s *t*-test.

^a^ Represents a significant difference between control and DIBC groups.

## Discussion

According to the WHO, the prevalence of obesity has more than doubled around the world in the last three decades. In 2014, there were more than 1,900 million overweight adults (≥18 years), and of these, over 600 million were obese [23]. In Mexico, the 2012 *National Survey of Health and Nutrition* (ENSANUT) reported that the prevalence of overweight and obesity in women were 35.5% and 37.5%, respectively, with a combined prevalence of 73%. In this study, we observed Mexican females, both control subjects and DIBC patients, who were overweight or obese; overall, 70% of the controls and 74% of patients belonged to the two principal risk groups for chronic, degenerative diseases, consistent with previously described data [27]. Several studies suggest that both WC and WHR (alone or combined) are more reliable markers than BMI to evaluate abdominal obesity or visceral adipose tissue (VAT), which is strongly correlated as a risk factor with diabetes mellitus, cardiovascular disease, cancer, and mortality [23]. In this work, we observed that HC, WC, and WHR measures were significantly higher in DIBC patients than in control subjects. Some studies have shown that for each 5 kg gain, breast cancer risk increases by 11% in postmenopausal women and that breast cancer patients have a strong tendency to gain weight after diagnosis. Some 33% gain weight the first 6 months and 56% in the 18 months after diagnosis, with the respective increases representing about 13% and 36% of the BMI [28,29]. Unfortunately, we could not determine whether the overweight status of our patients occurred before diagnosis or during the course of the disease. Weight increase in breast cancer patients after diagnosis could be due to age, menopause status, ER/PGR (oestrogen receptor/progesterone receptor) status, hormone replacement therapy, and other factors [30–32]. Although we did not explore menopausal and ER/PGR status, one or both may have influenced the adiposity of participants. We observed in the metabolic biomarkers that DIBC patients had higher values of glucose, whereas control subjects had increased concentrations of cholesterol; however, when we split our sample according to risk, the only significant difference among the two groups involved LDLc: the control group had higher values than the DIBC group. Possibly, changes in the patients’ lifestyles improved their serum adiponectin concentrations and fatty acid oxidation [30,33]. We previously assessed these same adiponectin SNPs polymorphisms in colorectal cancer patients but failed to find any association; however, the observed frequencies in both studies are similar to those found in other populations [34,35]. Because the association of adiponectin variants with several diseases, including breast cancer, is well known [18,36], we evaluated two of the variants with major clinical relevance in this work, namely rs2241766 and rs1501299. In rs2241766, the absence of the GG genotype in the breast cancer group supports the notion that TG and TG+GG genotypes confer protection against DIBC, a consequence perhaps related to the higher levels of serum adiponectin exhibited by carriers of the GG genotype [35,37,38]. In the rs1501299 variant, we observed that the T allele, even in heterozygotes, is a risk factor for breast cancer in our population. This result is comparable to those reported by Al Khaldi et al. (2011), who observed that carriers of the T allele and TT genotype had low levels of serum adiponectin. Contradictory results have been reported in different populations and breast cancer types. For instance, several studies did not find any association of the adiponectin SNPs rs2241766 and rs1501299 with the basal-like and luminal A breast cancer subtypes in African-American and other populations [38–41]. Certainly, it is possible that other environmental factors, such as exercise, modify the adiponectin concentration independently of the gene variants [42]. The discrepancies between our results and previous studies could be explained by ethnic differences, tissues analysed, sample size, and the different stages and types of breast cancer. In brief, we attempted to explore the relationship of some metabolic markers with breast cancer in order to create a reliable predictor panel.

However, limitations of our study, such as small sample size, unknown hormonal and triple marker status, treatment received, and the inclusion of only one specific breast cancer subtype, point to the need for a broader strategy in the Mexican population at risk.

Ductal infiltrating breast cancer (DIBC) is one of the most common cancers in Mexican women, and the identification of reliable biomarkers can help with prevention in the susceptible population. Our study suggest that Mexican women with hyperglycaemia and high measures of WC, HC and WHR may are at an increased risk for DIBC development. Additionally, the G allele and the TG and TG+GG genotypes of the *ADIPOQ* rs2241766 SNP appear to protect against DIBC development, whereas the T allele and the TT and GT+TT genotypes of the *ADIPOQ* rs1501299 SNP emerge as risk factors for this cancer in the Mexican population. These results highlight not only the importance of adiposity measures as risk factors for breast cancer in Mexican populations but also their association with only one variable of ADIPOQ (rs1501299 SNP). It is important to explore other gene interactions in order to reveal their effects.
